# The liberalization of fireworks legislation and its effects on firework-related injuries in West Virginia

**DOI:** 10.1186/s12889-020-8249-0

**Published:** 2020-01-30

**Authors:** Toni M. Rudisill, Katarina Preamble, Courtney Pilkerton

**Affiliations:** 1 Department of Epidemiology, West Virginia University, School of Public Health, PO BOX 9190, Morgantown, WV 26506 USA; 2 School of Public Health, West Virginia University, PO BOX 9190, Morgantown, WV 26506 USA; 3 Department of Family Medicine, West Virginia University, PO BOX 9152, Morgantown, WV 2506 USA

**Keywords:** Legislation, Policy, Injuries, Epidemiology

## Abstract

**Background:**

Fifteen states, including West Virginia, have liberalized their laws concerning fireworks possession and sale. Effective June 1, 2016, House Bill 2852 enabled all Class C fireworks to be sold within the state. The effects of this policy on fireworks-related injuries requiring immediate medical care are unknown. The purpose of this study was to determine whether this policy may have affected the fireworks-related injury rate and/or injury severity.

**Methods:**

Data were collected from the electronic medical records of patients treated by West Virginia University Medicine between June 1, 2015-May 31, 2017. The pre and post law periods were defined as June 1, 2015-May 31, 2016 and June 1, 2016-May 31, 2017, respectively. Fireworks-related injuries were identified via International Classification of Disease Clinical Modification codes and by free text searches of the electronic medical records. The rate of injuries pre and post-legislation were compared by Exact Poisson Regression, while demographic characteristics and injury severity were compared via Fisher’s Exact tests.

**Results:**

56 individuals were treated for fireworks-related injuries during the study period. The majority of patients were over 25 years of age (64%) and male (77%). Most of the injuries occurred within 7 days of a celebrated U.S. holiday (64%), and 28% were severe in nature. Age, sex, and injury severity did not significantly differ pre and post law passage. The injury rate per 100,000 patients was 39% higher after the law was enacted (*p* = 0.3475; incidence rate ratio 1.39, 95% Confidence Interval 0.74, 2.68).

**Conclusion:**

The law increasing access to Class C fireworks may have affected the injury rate, but not injury severity among treated patients. Effective, evidence-based, public health interventions applicable to all age groups may be warranted particularly around national holidays. This study may inform other states looking to amend their legislation.

## Background

According to the American Pyrotechnics Association, a firework is “any device activated by combustion, de-flagaration, or detonation that produces a visual and/or sound effect” [1]. Numerous types of fireworks exist ranging from devices that can be ignited on the ground to produce sound or smoke to large aerial light displays. While professional fireworks displays are often safe for public viewing, firework-related injuries are common with recreational consumer use [2]. Between 2006 and 2010, there were 25,691 emergency department visits in the U.S. attributed to firework-related injuries [3]. Another study reported that the number of firework-related injuries treated at emergency departments in the U.S. to be 97,562 between 2000 and 2010 [4] Among epidemiologic studies conducted within the U. S, most studies concur that males and children, especially those between 10 and 14 years of age, are the most commonly affected demographic groups [3–10]. While fatalities are infrequent, burns, contusions, and lacerations, mainly to the hands, arms, and eyes, are commonplace [3–10]. Fireworks can be dangerous because they are often unpredictable and the chemicals which comprise them can be highly variable [11]. Many fireworks-related injuries are caused by device misuse, improper handling, or device failure [12, 13]. Experimental studies have shown that many recreational fireworks do not detonate properly or in a timely fashion which may cause users to approach them prematurely [14].

To protect the public, fireworks are regulated by both federal and state laws. Fireworks are often classified by their type and amount of explosive material in which they contain; consumer fireworks are classified as Class C devices because they pose less of a threat to personal safety, while more hazardous, explosive materials are categorized as Class B devices [15]. Class B fireworks cannot be sold to the public, while states may regulate which type of Class C fireworks are permitted [15]. As of August 2016, 43 states and the District of Columbia allow some or all Class C fireworks, 4 states only allow sparklers (Illinois, Iowa, Ohio, Vermont), and 3 states ban all Class C fireworks to the public (Delaware, Massachusetts, and New Jersey) [14]. Since 2000, 15 states (Connecticut, Maryland, Minnesota, Vermont, Georgia, Arizona, Rhode Island, New York, Kentucky, Utah, New Hampshire, Maine, Michigan, Georgia, and West Virginia) have changed their legislation on fireworks possession and sale and most have chosen to lessen their restrictions [16]. West Virginia is the most recent state to amend their legislation. In March 2016, House Bill 2852 was passed. This legislation permitted virtually all Class C fireworks to be sold year-round to consumers within the state of West Virginia starting June 1, 2016.

Despite the trend to liberalize these laws, very few studies have investigated the effects of legislative changes on firework-related injuries in the U.S. [10, 15, 17] or even in other developed countries [18–20]. Most of the studies conducted in the U.S. have found that liberalized laws are associated with greater injuries and/or property damage [10, 15, 17]. Studies from the United Kingdom and New Zealand found restricted laws are associated with less injuries, while a study in Ireland found no effect [18–20]. Consequently, the purpose of this study was to investigate whether the liberalization of the firework law in West Virginia was associated with an increase in fireworks-related injuries requiring medical treatment and to determine whether the severity of these injuries changed as a result. The study hypothesis was that the injury rate would increase especially since the legislation was enacted prior to national holidays, such as the Fourth of July and Labor Day, which are commonly associated with firework use. It was also believed that injuries would be more severe as more types of fireworks could be sold throughout the year.

## Methods

### Data sources and collection

All data for this analysis were collected from the electronic medical records of eligible patients obtained via West Virginia University Medicine’s (WVUM) electronic medical records systems. WVUM is the largest healthcare system within West Virginia and has numerous treatment locations throughout the state. Data were collected through manual data abstraction and with the assistance of the West Virginia University Clinical and Translational Science Institute’s (WVCTSI) staff and Integrated Data Repository. The total number of unique patients and total number of patient visits by the WVUM health system within the pre and post law period were also collected. The population of West Virginia during both study periods was obtained from the U.S. Census Bureau [21]. This study and access to the data were approved by West Virginia University’s Institutional Review Board (protocol #1610302054R002).

### Study population

The sampling frame consisted of any individual who received treatment at a WVUM facility between June 1, 2015-May 31, 2017 for a fireworks-related injury. Firework-related injuries were identified via two sources: 1) by the International Classification of Disease, Ninth and Tenth Revision, Clinical Modification codes (ICD-9, ICD-10, respectively) E923.0-E923.9, W39XXXA and 2) by a free text search of patients’ entire electronic medical records for phrases and parts of phrases including, “firework, firecracker, class c, bottle rocket, rocket, roman candle, sparkler, sparkling devices, pyrotechnic, explosive, snap-cap, snappers, cherry bomb, trick noisemakers, missile type rocket, smoke device.” This combination of searching for patients via ICD codes and free text is far superior to searching by ICD code alone and has been shown to identify more patients for study [22].

### Variables

The primary exposure was the presence/absence of the law. Since the law became effective June 1, 2016, the pre-law period was defined as June 1, 2015 through May 31, 2016. The post-law period was defined as June 1, 2016 through May 31, 2017. The primary outcome of interest was the number and rate of fireworks-related injuries. The rates of fireworks-related injuries were calculated per capita, by the number of unique patients, and by total patient visits incurred during each time period.

Other covariates of interest included age, sex, injury severity, injury location, and whether or not the injury occurred within 7 days of a commonly celebrated U.S. holiday. The classification of these variables is presented in Table 1. Holidays included: New Year’s Eve, New Year’s Day, Memorial Day, Mother’s Day, Father’s Day, Fourth of July, Labor Day, Halloween, Thanksgiving, Christmas Eve, and Christmas Day. Injuries were classified into severity status at time of clinical presentation; categories of severity were mild to moderate, severe, or unclear. Severe injuries included those where the individuals suffered a first degree burn or greater over a large portion or their body (i.e. multiple appendages), a bone fracture/break, concussive-type injuries (i.e. traumatic brain injury), or amputation of an appendage. Mild to moderate injuries included lacerations, contusions, minor burns, a burn of any severity to one body part, eye injuries, sprains, and partial hearing loss. Injuries where it was difficult to determine severity were listed as unclear.

**Table 1:** Characteristics of individuals treated for fireworks-related injuries in West Virginia (*N* = 56)^a^

	Number	Percent	*P*-value^b^
Characteristic			
Age (years)^c^			0.3840
≤ 25	20	35.7	
> 25	36	64.3	
Sex			0.5104
Male	43	76.8	
Female	13	23.2	
Injury Severity^d^			
Mild-Moderate	36	72.0	0.7438
Severe	14	28.0	
Unclear/Missing	< 10		
Injury Location			
Eye	10	17.9	
Hand	18	32.1	
Other	28	50.0	
Holiday Period			0.5713
Yes	36	64.3	
No	20	35.7	
Law Status			
Pre-law injury	20	35.7	
Post-law injury	36	64.3	

^a^ Due to small sample sizes, demographic characteristics are not presented by pre and post law status to protect patient confidentiality

^b^ The *p*-value compares the demographic characteristic before and after the passage of the law by Fisher’s Exact test

^c^ Age range: 2–69 years; mean age = 33 years, median age = 32.5 years; mean age pre-law 29.7 years (standard deviation 14.5); mean age post-law 34.8 years (standard deviation 16.9)

^d^ Severe injuries included those where the individuals suffered a first degree burn over a large portion or their body (i.e. multiple appendages), a bone fracture/break, concussive-type injuries (i.e. traumatic brain injury), or amputation of an appendage

### Statistical analysis

Demographic characteristics were summarized via percentages and frequencies. Patients’ age, sex, and injury severity were compared pre and post law periods by Fisher’s Exact Tests. To determine whether the injury rate changed significantly after the law was passed, Exact Poisson Regression was utilized; this statistical methodology was chosen due to the small sample size [23]. The total number of unique patients, total number of visits, and resident population during the pre and post law periods were used as the variable offsets. To protect patient confidentiality, the demographic characteristics stratified by law status were not presented in tables. All analyses were conducted using SAS/STAT software, version 9.3 (Cary, NC) with a two-sided significance level (α = 0.05).

## Results

Over the study period, 56 patients sought treatment for fireworks-related injuries (Table 1). The majority of patients were over 25 years of age (64%) and male (77%). While most injuries were mild to moderate, 28% were severe in nature. The majority of severe injuries resulted in the amputations of appendages. Most injuries occurred to the hands (32%) and eyes (18%). Most injuries occurred within 7 days of a celebrated U.S. holiday (64%), but mainly around the Fourth of July. Age, sex, and injury severity did not significantly differ pre and post law passage (*p*-values: 0.3840, 0.5104, and 0.7438, respectively). The majority of injuries occurred after the law was passed (64%). Regardless of the denominator used, the rates of injury were up to 51% higher after the law passed (Table 2).

**Table 2:** Firework-related injury rate pre and post-law passage per 100,000^a^

Denominator	Pre-law Rate	Post-law Rate	Rate Ratio^b^	95% CI	*P*-value^c^
Unique Patients	4.7	6.6	1.39	(0.74, 2.68)	0.3475
Healthcare Visits	0.73	1.0	1.38	(0.73, 2.67)	0.3576
Per Capita	0.98	1.47	1.51	(0.80, 2.91)	0.2256

*Abbreviations*: *CI* Confidence Interval

^a^ Injury rates are presented by various denominators including by the number of individual/unique patients seen by West Virginia University Medicine, the number of total healthcare visits, and per capita rates during the study periods. All rates presented are per 100,000 individuals. ^b^ The referent group is the pre-law rate. ^c^ The *p*-value was calculated via Exact Poisson Regression

## Discussion

There were two principal findings as a result of this analysis. First, this study found that the fireworks-related injury rate appeared to increase among patients treated by West Virginia’s largest health system after the passage of the liberalized fireworks law. While these pre and post law rates did not statistically differ, this could be attributed to the small sample size. Additionally, the severity of the injuries at time of clinical presentation did not appear to change after the law’s enactment. These findings suggest that as firework accessibility increased, more individuals were exposed to these objects and increased their risk of injury. Secondly, the demographic population affected was older than anticipated. It is commonly accepted in the injury literature that youth often engage in riskier behaviors than most adults. Thus, these findings may inform future public health prevention or policy measures.

The peer-reviewed literature regarding fireworks-related injuries in relation to legislation in the U.S. is sparse. Four studies have investigated the effects of fireworks legislation on fireworks-related injuries in the U.S. previously [5, 10, 15, 17]. Three of these studies had methodological issues/concerns which may limit their credibility and utility for investigating this issue [5, 15, 17]. However, there was one, methodologically sound, prospective study that was conducted in Washington State in the 1980’s. That study investigated fireworks-related injuries among 11 participating hospitals before and after legislation was passed in the state; this particular law permitted the state-wide sales of fireworks [10]. Previously, fireworks sales were only permitted on Native American reservations. That study found that after access to fireworks was increased, there was a 125% increase in fireworks-related injuries collectively in the 11 hospitals. Thus, these findings are similar, but not as pronounced, as those identified in the present study [10].

Virtually all other studies conducted in the U.S. regarding fireworks injuries have been descriptive in nature; moreover, most have primarily focused on injuries incurred by pediatric patients [3, 4, 6–9, 24]. These studies concur that males sustain fireworks-related injuries more often than females and that these injuries often occur around national holidays, particularly the Fourth of July [3, 4, 6–9]. This coincides with the findings from the present analysis as well even though fireworks can be sold year round in West Virginia. One slight difference seen with the present analysis is that the study population was older than expected with 64% of the injuries occurring among individuals over 25 years of age. One previously conducted national study found that 49% of patients treated for fireworks-related injuries were over 20 years of age [3]. Thus, these findings suggest that these types of injuries may not just occur among adolescents. Middle-aged adults may actually be at a greater risk for these types of injuries. It is possible that adults may prohibit children from playing with more dangerous Class C fireworks, such as aerial rockets, but may personally use them instead; this may explain why there was a slight change in patients’ age pre and post law passage.

Collectively, these findings may have distinct public health and policy implications. Fireworks-related injuries often affect patient’s eye sight, hearing, cognition, and/or mobility. These injuries can be very traumatic, expensive, or chronic in terms of treatment. Severe injuries can lead to permanent disability and may require extensive physical and occupational therapy or pain management. Thus, states looking to amend their legislation may want to consider these ramifications. Also, these injuries do not just occur in children or adolescents and are common among adults. While proven interventions to prevent these types of injuries is lacking from the extant literature, effective interventions may be needed to address all age groups.

### Limitations

While this study utilized a comprehensive search strategy to identify injured patients treated by West Virginia’s largest healthcare system, it is not without limitation. First, this study was limited to a pre-post law analysis. While comparing these findings with a “control” state would have been preferred, the authors did not have access to medical record data from West Virginia’s neighboring states. Our search strategy was more detailed as we could search diagnostic codes and free text to identify potential cases. Additionally, it is entirely possible that some patients were not identified and subsequently excluded from the analysis as there are inherent limitations associated with medical record reviews. For example, the completeness of the record is reliant on hospital staff and by the reporting of patients or their legal guardians. Thus, records may not be entirely complete, accurate, or highly detailed. The sample size was small so there may not have been enough statistical power to detect difference between the study periods. A power analysis revealed that over 215 patients would be needed to detect a statistical difference between both pre and post law periods (e.g. two-tailed Poisson regression, exponentiated β1 = 1.3, α = 0.05). Because of sample size, we also could not adjust the models for any potential confounding variables. Moreover, it was also assumed that the patients purchased fireworks within the state of West Virginia; this information was unknown and was not included in the medical records. Additionally, while WVUM is the states’ largest healthcare system and has numerous treatment locations across the state, these findings were limited to the catchment area of one health system. Thus, the findings may not be completely generalizable to the entire state. However, WVUM has over 1.4 million patients and is located in a state with a population of ~ 1.8 million residents. It is possible that some individuals sought treatment at healthcare facilities outside of the WVUM network. Additionally, it is possible that individuals may have been injured by fireworks, but decided not seek medical treatment even though it may have been warranted. Lastly, injuries were classified by severity at time of clinical presentation, which may differ from the impact of the injury overtime. It is difficult to gauge how deeply an individual was impacted by their injury after they received medical care even if it appeared less severe compared to others.

## Conclusions

The present study found that fireworks-related injuries increased among patients treated by West Virginia’s largest health system after House Bill 2852 became effective. This particular piece of legislation permitted the sale of Class C fireworks within the state. By increasing access to fireworks, the injury rate may have increased because the population was more exposed to these devices. Effective, evidence-based, public health interventions may be warranted not only for adolescents, but also for adults, around national holidays celebrated within the state.

## Data Availability

The data cannot be shared to protect patient confidentiality.

## Abbreviations

ICD: International Classification of Disease;
U.S.: United States
WV: West Virginia
WVUM: West Virginia University Medicine
